# A Treatise of Midwifery, Comprehending the Management of Female Complaints, and the Treatment of Children in Early Infancy. To Which Are Added, Prescriptions for Women and Children, and Directions for Preparing a Variety of Food and Drinks, Adapted to the Circumstances of Lying-In Women. Divested of Technical Terms and Abstruse Theories

**Published:** 1781-03

**Authors:** 


### LONDON MEDICAL JOURNAL,

For MARCH 1781.

### SECTION I.

### BOOKS.

I. *A Treatise of Midwifery, comprehending the Management of Female Complaints, and the Treatment of Children in early Infancy. To which are added, Prescriptions for Women and Children, and Directions for preparing a Variety of Food and Drinks, adapted to the Circumstances of Lying-in Women. Divested of technical Terms and abstruse Theories. By* Alexander Hamilton, *Professor of Midwifery in the University of* Edinburgh, *and Member of the Royal College of Surgeons.* 8vo. Edin. 1781.

THE art of midwifery has been retarded in its progress by a variety of circumstances. For many ages it was entirely confined to women. The timidity and delicacy peculiar to the female character would, no doubt, for a long time prevent the interference of the other ſex; but at length, the refinements of faſhion, and the unreſerved connection between the two ſexes, weakened this powerful obſtacle; ſo that the arguments derived from this amiable but miſtaken modeſty, yielded by degrees to the love of life, the peculiar tenderneſs of the mother, and the affection of the wife; and male practitioners were employed to give that aſſiſtance for which their improved knowledge, their courage, preſence of mind, and frequently their bodily ſtrength, had particularly qualified them. It muſt be allowed, however, that when this change took place, the accoucheur uſually attributed too much to art, and relied too little upon nature; but this diſtruſt of nature was owing not ſo much to the fault of the artiſt as to the imperfection of the art. We are now more fully informed of the ſeveral circumſtances which juſtify our interference, or lead us to an exact, patient attendance on the efforts of nature.

Theſe preliminary obſervations are extracted chiefly from the preface to the work before us, which, though profeſſedly written for the inſtruction of midwives, and on that account diveſted of technical expreſſions, is well deſerving the attention of the moſt experienced male practitioner.

Mr.

Mr. Hamilton firft gives a concife general view of the female anatomy. After which he treats feparately of the pelvis both in its natural and diftorted ftate; of the ftructure and figure of the child's head, of the foft parts of generation, the theory of generation, menfes, flooding, and the difeafes of the genital parts.

In fpeaking of the menfes our author obferves, that notwithftanding all that has been faid concerning emmenagogues, there is not, as yet, in the whole catalogue of medicines any one which can be relied on for that purpofe. He thinks that aloes owes its great character in promoting the menfes, merely to its violent operation and ftimulating quality. In conftitutions fubject to the piles, from the tenefmus or ftraining it occafions in going to ftool, it very often brings on that difeafe; in the fame way it may have a tendency to bring down the menfes: hence it is extremely improper in delicate fyftems and in women fubject to floodings. All ftrong purgatives, adds Mr. Hamilton, will act in the fame manner.

If purgatives fail, he recommends a fpoonful of white muftard feed, or a fmall cupful of a weak infufion of horfe-radifh twice a day, as no contemptible remedies. He remarks, that mecines given to promote the menfes, fhould be administered about a week before the expected return, and continued for a few days after, or till the ufual evacuation recurs; and that thefe, or an infufion of penny royal, tanfey, balm, or chamomile, may alfo be ufed with advantage when the difcharge is fcanty or fparing.

The author obferves, that *painful menftruation* chiefly happens to women of a delicate nervous habit, and to women of fafhion. Thofe of a lower clafs, who are inured to hard labour, are feldom obferved to fuffer at thefe times, unlefs from a difeafed ftate of the uterus. In cafes of this fort, he recommends half a grain or a grain of opium to be taken evening and morning till the menftrual period is over. The binding quality of the opiate is to be counteracted by the ufe of gentle laxatives or glyfters. He adds, however, that thefe indulgencies fhould be allowed only upon emergencies, as they are with difficulty left off.

In flooding, or an immoderate difcharge of the menfes, he thinks that more is to be expected from regimen than medicine. He confiders light, nourifhing, and cooling diet, cold drink, cool air, and cold applications (fuch as cloths foaked in vinegar and water) to the loins, abdomen, men, and os externum, as the principal remedies. He relies but little on the power of medicine. If the patient is of a full habit, hot and feverifh, he gives nitre; if otherwife, he prefers rofe tea acidulated with fpirit of vitriol. Alum whey is fpoken of as a powerful remedy. When there is much pain or anxiety, and no inclination to vomiting, he thinks that opiates may be given with advantage. He directs the belly to be kept open by the ufe of emollient glyfters, exhibited in a degree of heat, which we call tepid, that is, fcarcely milk warm. A light decoction of Peruvian bark, fharpened with elixir of vitriol, is recommended as the beft medicine to ftrengthen the fyftem, and prevent a return of the diforder.

Speaking of the final ceffation of the menfes, Mr. Hamilton obferves, that the morbid fymptoms which occur at this period, are rather to be afcribed to a general change of the habit, than merely to the abfence of the menftrual evacuation. He finds, that the women who are the moft apt to fuffer at the decline of life, are thofe who have never had children; who have never enjoyed good regular health; whofe health has been impaired by frequent labours or mifcarriages; who have been fubject to irregularities ties of the menfes, to the fluor albus, or to nervous and hyfterical fymptoms. He remarks, however, that frequently, delicate women who have been diftreffed with painful menftruation, or with nervous complaints while regular, gradually recover after this period, and for a long time enjoy a ftate of health to which they were before ftrangers.

The fymptoms that appear about this time are arranged by our author under the following heads, viz. 1. Thofe of fulnefs, in confequence of the fudden ftoppage of an ufual evacuation in full habits; 2. Frequent, long-continued, or immoderate floodings in feeble relaxed habits; 3. General affections of the fyftem from an alteration of the conftitution. The complaints of the firft of thefe claffes are to be relieved by fpare diet, occafional venæfection, avoiding coftivenefs, and by exercife. Thofe of the fecond clafs are to be treated in the manner juft now directed; but if the difcharge is owing to fulnefs, proper evacuations will be neceffary. In cafes of the third kind, when fhooting pains appear about the under part of the abdomen or region of the uterus, and in the breafts, with other fymptoms of bad health, they evidently indicate a change in the conftitution, which depends depends on other circumſtances than the cloſing of the veſſels of the womb, and the mode of treatment muſt vary accordingly. Our author thinks it right however to remind the reader, that as the womb is acutely ſenſible, the firſt ſymptoms of the diſeaſe often ariſe from it; that thoſe parts firſt ſuffer that are moſt immediately, by nervous ſympathy, connected with it; and that, ſoon after, the general health becomes affected.

In treating of the diſeaſes of the genital parts, our author ſpeaks of the fluor albus as occurring, perhaps, more frequently than any other female complaint. He thinks it may often be diminiſhed, though ſeldom entirely cured, unleſs in young people, when the complaint is recent, by ſtyptic or aſtringent injections; ſuch as a weak ſolution of alum, or ſugar of lead in water, or Tunbridge water: when the fluor albus, as is often the caſe, is connected with the ſtate of the ſtomach, he finds the Peruvian bark, infuſed in lime water, one of the beſt remedies. In many inſtances he has had occaſion to obſerve, that women have been cured of the moſt obſtinate habitual fluor albus, by giving ſuck. He conſiders long-continued, or exceſſive fluor albus, when combined with

ſmallneſs

ſmallneſs of the breaſts, and irregular and ſparing or deficient menſes, and the appearance of extreme delicacy, as the moſt certain indication of ſterility.

Our author next proceeds to treat of pregnancy. He firſt traces the progreſs of the fœtus, and then conciſely deſcribes the other parts of the ovum; after which he points out the changes the uterus undergoes during pregnancy, and remarks, that as the contents of the uterus in early geſtation are entirely confined to the fundus, or upper part, the firſt change from pregnancy ariſes from the uterus ſinking downwards towards the lower circumference of the pelvis, by which means the abdomen is ſomewhat diminiſhed in ſize and appears flatter; and this circumſtance, when combined with the uſual ſymptoms of breeding, our author conſiders as a more probable indication of pregnancy than any other that can be depended on in the early months. He obſerves, that the uterus, when diſtended, becomes of a ſofter and more ſpongy texture; ſo that by unſkilful attempts to turn the child, or to ſtretch the orifice of the womb, it has often been torn, and the unfortunate woman has fallen a victim to the raſhneſs of an ignorant operator. He remarks, marks, that even the judicious Dr. Smellie was not aware of the dreadful confequences of anticipating nature in her operations; for he candidly acknowledges, that by attempting too early to dilate the orifice of the uterus in order to turn the child, the uterus was frequently torn; and although the woman fometimes recovers, where the thin membranous edge of the orifice only is torn, lacerations of the body of the uterus are always fatal.

The womb, during pregnancy, being chiefly enlarged towards the fundus, the ligamenta lata are left much below the principal bulk of the uterus; we are therefore very judicioufly cautioned not to pull violently at the umbilical cord to deliver the placenta, left we fhould occafion an inverfion of the uterus, or by not giving it time to contract, bring on a fatal flooding.

Our author contents himfelf with juft mentioning the ridiculous notion of fuperfœtation; and after fpeaking in the fame curfory manner of extra-uterine conception and monfters, proceeds to fpeak of the difeafes of pregnancy, which, though troublefome, he obferves are very feldom fatal.

 Amongft

Amongſt other ſymptoms of breeding, he enumerates, 1. *Sickneſs* and *vomiting:* theſe, when accompanied with marks of plethora, may be relieved by bleeding and a ſpare diet. When the ſickneſs is exceſſive, vomits of ipecacoanha have often the beſt effects; and it will ſometimes be neceſſary to repeat them once a week or oftener. Sometimes, however, the ſickneſs attending pregnancy is merely a nervous affection, and no ſenſible relief will be afforded till the uterus riſes above the brim of the pelvis. 2. This ſymptom, in ſome inſtances, accompanies all the ſtages of pregnancy: it may be palliated by attending to the ſtate of the ſtomach. 3. *Diarrhœa:* this may be remedied by gentle vomits, rhubarb, opiates, and regimen. 4. *Swelling and pain in the breaſts:* when theſe are exceſſive, and the patient is young, venæſection becomes neceſſary: tight preſſure ſhould be avoided, and the breaſts rubbed with warm fine olive oil twice a day, and afterwards covered with ſoft flannel or fur. 5. *Fainting, nervous, or hyſteric fits:* in theſe caſes the only certain remedy is opium.

In the more advanced ſtage of pregnancy, the preſſure of the uterus occaſions other complaints which often threaten the life of the patient, patient, whereas the former ones end only in miſcarriage. In noticing the ſymptoms that occur at this period, our author takes occaſion to ſpeak of the *retroverſion of the uterus*, a diſeaſe which till of late hath been but little attended to. No complaint immediately attending pregnancy requires ſo much attention as this. In the beginning, under proper management, there can be no hazard; but if neglected, the utmoſt danger is to be dreaded; for if the urine cannot be drawn off and the tumour reduced, death will be the unavoidable conſequence. It chiefly occurs from the third till the end of the fifth month. Thin ſpare women are the moſt ſubject to it.

In ſpeaking of the floodings that ſometimes come on in the advanced ſtage of pregnancy, he obſerves, that the moſt dangerous are thoſe where the placenta is attached at the neck or over the mouth of the uterus. The filling the vagina with tow, ſoaked in ſtyptic liquors, is a practice which, in the opinion of our author, has no particular advantage to recommend it; on the contrary, he thinks they may do harm by their irritation.

Mr. Hamilton briefly enumerates the cauſes of abortion, and points out the proper treatment in cafes of mifcarriage. He obferves, that in cafes of twins or triplets, one conception may be interrupted by the growth of another, and the fœtus perifhing, its ovum may be retained for fome time afterwards, and then mifcarriage, or the expulfion of that ovum, will enfue. The remaining conception may, however, be retained, and the woman, under proper management, be enabled to carry the child till full time.

With the generality of fyftematic writers, our author divides labours into the natural, laborious, and preternatural. He firft defcribes the progrefs of a natural labour, and obferves, that the *fpurious pains* (which are to be carefully diftinguifhed from the true ones) are frequently a prelude to approaching labour. He remarks, that labour pains often continue from fix to twelve, eighteen, or twenty-four hours; that is, if the woman be not delivered in fix hours, the labour will, perhaps, be protracted for fix hours more; if not in twelve, that fhe will then go on nearly to the end of the eighteenth hour, or to the twenty-fourth; and that every fix hours of pain generally alternate more or lefs with intervals of eafe. We much doubt whether this obfervation is well founded; and indeed the author thor thor himſelf acknowledges that the duration of labour is ſo precarious, that we ought to be cautious in giving any opinion. Speaking of the poſition for delivery, he obſerves, that when the labour proves tedious, the woman ought not to be confined long in any poſture.

He diſtinguiſhes three ſtages in a natural labour. The firſt is completed, when the os uteri is ſo much dilated that no part of the orifice can be felt. This is generally accompliſhed in about eight, ten, or twelve hours. During this ſtage, he very properly cautions the midwife againſt frequent touching; and obſerves, that it is unneceſſary to place the patient in the proper ſituation for delivery, till the os uteri is dilated to the breadth of half a crown. The delivery of the child, after the paſſages are dilated, conſtitutes the *ſecond ſtage* of labour. During this ſtage, the midwife is directed to examine at the time of every pain, as it may then be done without any inconvenience to the patient. The delivery is to be truſted wholly to nature, as the ſhoulders will generally accommodate themſelves to the ſhape of the pelvis, and turn towards the pubes and ſacrum. The *third ſtage* is the delivery of the placenta. The proper time for doing this is, when the contracting uterus has ſhifted its poſition, and can be perceived like a hard round ball, at or below the navel; or, when the woman complains of a grinding or griping pain. In moſt caſes, this happens from ten minutes to half an hour after the delivery of the child. The placenta adheres moſt firmly in premature births, when the woman has been in bad health during pregnancy; in lingering or difficult births; or when haſty attempts are made to extract it. It is moſt eaſily and quickly ſeparated in a firſt birth, when the woman is in good health, and when the labour has been properly managed. In floodings after delivery, the placenta is to be immediately delivered. In caſes of retention from the uncommon adheſion of the cake, the utmoſt caution is requiſite. When it is diſeaſed, and adheres to the uterus like moſs to a rock, force muſt never be uſed. Fortunately this caſe does not often occur. Mr. Hamilton obſerves, that adheſions of the placenta from diſeaſed ſchirroſity, always threaten ſome degree of danger, as the ſeparation may be productive of a fatal hæmorrhage.

Our author diſtinguiſhes the ſecond claſs of labours into, 1. The tedious or lingering; and 2. The difficult or laborious. The former, he obferves, may be owing to obftacles, arifing from the mother, the child, or the membranes, water, chord, or placenta. In the mother, tedious labours may proceed from general or local complaints. The general complaints may be, 1. *Cholic*, which may be palliated by clyfters and opiates. 2. *Naufea* and *vomiting* : thefe fometimes occur in the eafieft labours, and are generally the effect of fympathy. In thefe cafes warm water is directed to be drank freely. 3. *Flooding* : as the labour pains increafe, this fymptom generally abates. If it fhould not, we ought to promote the contraction of the uterus by breaking the membranes, when the os uteri is dilated about the breadth of a half-crown piece. This expedient, we are told, feldom fails to give an immediate check to the flooding. If the difcharge proceeds from the feparation of part of the placenta attached to the neck or over the orifice of the uterus, the flooding will increafe with labour pains, and there is no other method of preferving the woman and child, but by an expeditious delivery. 4. *Cramps* : thefe are fometimes relieved by opiates. 5. *Lownefs and faintnefs* : thefe accompany the firft part of labour only. When the ftrong pains come on, the woman recovers her her ſpirits, and acquires vigour and reſolution. 6. *Convulſions:* theſe may ariſe from various cauſes. Bleeding, laxative clyſters, and cool air, are the chief remedies. When it can be eaſily done, delivery ſhould be aſſiſted. 7. *Feveriſh indiſpoſition from fulneſs.* 8. *Hectic or conſumptive habit:* in theſe melancholy caſes the pains are weak and trifling; but as the os uteri affords but little reſiſtance, the child readily obtains a paſſage. Mr. Hamilton remarks, that hectic women, under proper management, rarely ſink immediately after delivery; they generally live a week or longer, though they ſeldom outlive the month. 9. *Paſſions of the mind.* 10. *Improper treatment:* under this laſt head, the author takes occaſion to caution us againſt exhauſting the patient's ſtrength in the beginning.

The *local* complaints retarding the progreſs of labour may be, 1. *Narrowneſs or diſtortion of the bones of the pelvis:* Mr. Hamilton ſuppoſes, narrowneſs at the brim to be the moſt frequent defect of the pelvis, and the moſt difficult to diſcover, as we can only aſcertain it by the ſymptoms; for we ſhould not attempt to introduce the hand, till the os uteri is dilated, 2. *Thickneſs and rigidity of the os uteri:* this, we are told, is one of the moſt common cauſes of lingering labours. It chiefly occurs in elderly women, in ſtrong robuſt conſtitutions, or where the intervals between child-bearing have been diſtant. In a firſt labour, or when a woman is advanced in life, from thirty-ſix hours to three days may be required for the dilatation of the os uteri; yet if the management be properly regulated, neither the mother nor the child will ſuffer from this delay. Other local complaints retarding labour may be dryneſs and conſtriction of the vagina—a diſeaſed ſtate of the parts—ſwelling or inflammation of the vagina—prolapſus of the uterus, vagina and rectum—ſtone in the urethra—hardened fœces.

The author obſerves, that when the protraction of labour depends on the child, it is uſually occaſioned either by the bulk or ſolidity, or the unfavourable poſition of the head. He tells us that a large head may be ſuſpected when the vertex does not lengthen out by the force of the pains, and when the progreſs of the labour is ſuſpended, though the pains continue to be ſtrong and frequent after the ſoft parts are ſufficiently dilated. The unfavourable poſitions of the head, he thinks, may be referred to two kinds, which include a conſiderable variety; 1ſt, When

 the the crown inftead of the vertex prefents; 2dly, Face cafes. He obferves, that in cafes of the firft fort the labour is generally more tedious, and the hazard of lacerating the perinæum greater than when the vertex prefents, but that if no other obftacle occurs, the labour will commonly terminate favourably if left to nature. Our author remarks, that of all laborious births, face cafes are the moft difficult. The varieties of thefe cafes may be known by the direction of the chin. In every cafe of this fort, his rule is to allow the labour to go on till the face is protruded as low as poffible.

The placenta and its appendages may retard labour, by the membranes being too weak or too ftrong, by the waters being too copious or too fparing, by the chord being too fhort or too long. The placenta itfelf may likewife be improperly attached over the os uteri.

Our author gives the appellation of *difficult*, or *ftrictly laborious labours*, to cafes that require the ufe of inftruments; but as his work is defigned chiefly for the female practitioner, he fays but little on this fubject, and therefore proceeds to fpeak of the third clafs of labours, the preternatural. In all cafes of this fort, the delivery, he obferves, is always precarious and often difficult.

difficult. In delivering rules for turning the child, Mr. Hamilton remarks, that when the hand is within the pelvis, and there is a neceſſity for paſſing it pretty high in the uterus, to ſearch for the child's feet, the proper direction is not preciſely in the line of the navel, as Dr. Smellie adviſes, but inclining it a little to one ſide, to avoid the prominent angle of the joints of the lumbar vertebræ at the upper part of the ſacrum, by which more room will be gained, and leſs pain given to the woman; for the uterus preſſes ſtrongly there.

Mr. Hamilton divides preternatural labours into four claſſes. In thoſe of the firſt claſs one or both of the lower extremities preſent. In thoſe of the ſecond, the child lies acroſs the pelvis, with the arm, ſhoulder, ſide, back, or belly preſenting. In thoſe of the third, one or both arms are protruded before the head. The fourth claſs conſiſts of premature or flooding caſes, or of thoſe in which the navel ſtring falls down double before the preſenting part, and the child's life is in danger from its compreſſion. Examples are given of each of theſe claſſes; after which the author deſcribes the method of delivery in flooding caſes, and in caſes of twins or triplets.

He next treats of the management of women after delivery. He thinks, that a few plain rules, fuggefted by common fenfe, and a careful attention to the dictates of nature, are in moft cafes fufficient. He obferves, that it is neceffary to attend, 1ft, To the regulation of the body; 2dly, To that of the mind. He inculcates the neceffity of cool air and regimen, and of the occafional ufe of clyfters and gentle laxatives to obviate coftivenefs. He thinks the patient fhould be taken up, and have her bed properly adjufted on the fourth, or at the lateft, the fifth day after delivery, and that her fhift, &c. fhould be changed once a day or oftener.

Mr. Hamilton next treats in a curfory manner of the accidents that happen in confequence of delivery, fuch as fwelling of the labia, laceration of the perinæum, inflammation, abfcefs or gangrene of the genital parts; rupture of the vagina, laceration of the uterus, and inverfion of the uterus. In fpeaking of the latter he obferves, that of five inftances where it happened from the ignorance of the practitioner in hurrying the extraction of the placenta, one lady only furvived the dreadful accident. Her recovery is the more extraordinary, as the uterus could not be reftored to to its natural ſtate, and though replaced within the vagina, it ſtill continues inverted.

After theſe obſervations follow ſome remarks on the diſeaſes incident to a child-bed ſtate, ſuch as faintings, floodings, after-pains, inflammation of the womb, and irregularities of the lochia. Mr. Hamilton obſerves, that as inflammation, excoriation, coalition of the labia, and even of the os uteri, have been produced by a ſtagnation of the putrid lochia, lying-in women ought to be very careful to keep theſe parts clean, by frequent bathing with a ſponge and warm water, while the lochia continue to flow; and ſhould afterwards take a proper opportunity, when their health will permit, of applying cold water, or of uſing the cold bath, when the ſeaſon and other circumſtances will admit of it. " The " advantages, adds our author, of obſerving a " ſcrupulous cleanlineſs at theſe times, and after " *menſtruation*, though little attended to in this " country, are ſufficiently obvious, and do not " require any other arguments to enforce it.

" The practice of ablution was firſt known " among the ancient Jews, and conſtituted a part " of their religious ceremony. It was probably " firſt ſuggeſted by delicacy, and afterwards " eſtabliſhed on account of health. It ſtill pre-

" vails

" vails in the eaftern countries; and the *biddeau* " of the Italian and French ladies deferves the " imitation of thofe of Britain, who in general " furpafs moft other nations in delicacy of fen- " timent, if not in politenefs of manners."

The author next fpeaks of the determination of fluids to the breafts, and its confequences. He remarks, that from the third to the fifth day after delivery is a very important period; for in this interval the red *lochia* ceafe, and the dif- charge is only compenfated by the *milk*, which generally flows in full ftreams. Difeafes may therefore arife from its being too full or too fparing.

When the colour of the lochia begins to change, pains in the lower part of the abdomen, like thofe of painful menftruation, come on, attended with a pretty fmart fever, and at length the breafts become enormoufly diftended, and occafion the moft violent pain, weight, and throbbing. This febrile commotion and painful tenfion continue from twenty-four to thirty-fix hours, and are commonly terminated by a fweat, diarrhœa, or free difcharge of milk. When the woman is healthy, is to fuckle her child, and has good nipples, the author advifes us to put the child to the breaft within twenty-four hours from delivery.

delivery. In repelling the milk where this is thought requiſite, he truſts wholly to an abſte-mious diet for ſome days, with little drink, keep-ing the body gently open, and rubbing warm oil on the breaſts two or three times a day. He obſerves that the ſtructure of the breaſts of wo-men is more complicated than in any other claſs of animals, the lactiferous tubes being larger and more ſtrait, ſo that the milk cannot flow out in-voluntarily.

He diſtinguiſhes two kinds of abſceſſes of the breaſts; thoſe that are ſeated deep in the glan-dular ſubſtance of the breaſt, and thoſe that are more ſuperficial. The former are tedious in their progreſs to ſuppuration, painful, and at-tended with fever: the latter are much milder, burſt ſpontaneouſly, and the ſore heals kindly. Mr. Hamilton obſerves, that in thoſe of the firſt ſort, the woman may be ſaved much pain by opening the tumour early. He remarks, however, that ſuppuration often returns in other parts of the breaſt two or three different times.

In ſpeaking of ſore nipples, the author ob-ſerves, that women who have been ſubject to a complaint of this ſort ſhould endeavour to pre-vent its return by applying aſtringents to the parts for ſeveral weeks before delivery, as cloths dipped in alum water, in ſtrong ſpirits, or in the brine of ſalted meat boiled up.

Beſides the milk fever, Mr. Hamilton treats of the *weed*, the *miliary*, and the *puerperal fever*. The firſt of theſe, the *weed*, he defines to be a fever in the child-bed ſtate, occaſioned by miſmanagement or accident, and happening chiefly to irritable patients. It differs, we are told, from other fevers in the violence and duration of the cold fit, and is generally terminated in twenty-four hours. It is ſeldom dangerous, but leaves the woman liable to future attacks. In the cold fit tepid diluent drinks are recommended; in the hot fit cool drinks and cool air. The ſweating is not to be protracted too long, or too ſuddenly checked. The body is to be kept open by means of glyſters.

Our author ſuppoſes the miliary eruption to be conſtantly a ſymptomatic diſeaſe, the effect of a hot regimen, in lying-in women. He obſerves that women in this ſtate are much prediſpoſed to putrid diſeaſes, ſo that when a ſweat is urged, the putrid matter exiſting in the ſyſtem will be driven to the ſurface, and when the quantity is unuſually large, and the quality preternaturally acrid, it will not only be poured out in a greater quanquantity than the pores of the outer ſkin can admit to paſs, but ſtagnating under it, will induce an inflammation and eruption.

Mr. Hamilton conſiders the diſeaſe in its mildeſt ſtate as being of a nervous or putrid nature; and obſerves, that the danger is increaſed, as the diſeaſe is complicated with other complaints. During the anxiety, we are directed to open the ſeveral excretories, particularly thoſe of the ſkin; but after the eruption appears, we are to endeavour to regulate the determination, and carry it on as ſlowly as poſſible. The remedies recommended in the former of theſe ſtates are vomits, laxatives, and fomentations of a moderate warmth to the legs and thighs; in the latter, nitre, cool acid drinks, ripe fruit and cool air. In caſes of great debility, or when putrid ſymptoms come on, the Peruvian bark, and the moderate uſe of wine, are deemed neceſſary.

Our author deſcribes the *puerperal fever* as coming on "generally about the ſecond or "third day after delivery, attended with conſi- "derable debility, a ſoreneſs of the head, "chiefly confined to the forehead, and fre- "quently with vomiting." This definition, he thinks, will diſtinguiſh it from every child-bed

 diſeaſe, difeafe, except perhaps, the miliary fever; the nature of which, in doubtful cafes, will be foon apparent. Though the puerperal fever commonly occurs about the evening of the fecond day, it in fome inftances, we are told, comes on fo late as the fifth or fixth day. Its duration is various; but the eleventh day is the moft frequently critical. Mr. Hamilton obferves, that the immediate caufe of this fever is ftill involved in much obfcurity; it frequently occurs after the moft eafy and natural delivery, and where no particular caufe can be affigned. The moft common occafional caufes he thinks are, probably, improper management during pregnancy, in time of labour, and after delivery. He obferves, that it is remarkably infectious, and its event in general fo fatal, that like the plague, few efcape of thofe affected. In 1774 it appeared in the lying-in ward of the Edinburgh Infirmary, and its event in moft cafes was fatal. But it has never occurred there fince, and is very little known in private practice. The author adds, that if any means can prevent it, they will chiefly confift in a ftrict obfervance of cooling regimen, free air, and cleanlinefs; and when the difeafe fhews its pre-

ſence, we muſt proceed in the treatment on the general principles of putrid fevers.

The author next proceeds to treat of the management of children. He obſerves, that it would be ſuperfluous, perhaps, to attempt to ſhew, that the mortality of infants, which aſtoniſhes and diſtreſſes every humane and intelligent inquirer, has in a great degree ariſen from miſtaken views. He points out the neceſſary attention to cleanlineſs, clothing, the evacuation of the meconium, nutrition, air, exerciſe, &c. He remarks, that children ſhould be waſhed every day till they are ſeveral years old, and that after the firſt week they ſhould be plunged every morning into cold water. Their clothing ſhould be light and ſimple; tape ſhould be uſed inſtead of pins, and every neceſſary precaution taken to prevent wet and dampneſs. Speaking of nurſing, the author obſerves, that children ought either to be weaned before the period of teething commences, or not till the danger from teething is over. He thinks it beſt to deprive the child of the breaſt by degrees. He conſiders the regimen of nurſes of great conſequence, though little attended to. One great motive that induces poor women to ſubmit to the drudgery of becoming nurſes for others, is with a view of living better; but our author very properly obſerves, that women ſuddenly tranſported from miſery and wretchedneſs to high life, that is, from poverty and activity to luxurious living and indolence, are very improper for the office of nurſing. It ought, therefore, to be a rule, to confine them as near as poſſible to their uſual diet and manner of life, or to introduce a change very gradually.

Mr. Hamilton obſerves, that it is unuſual for a woman to menſtruate while giving ſuck, but it ſometimes happens; and in ſuch caſes, the child ſuffers a ſlight indiſpoſition for a day or two before the menſtrual flux of the nurſe appears; but afterwards, no inconvenience ſeems to follow.

He divides the diſorders incident to new-born children into, 1. Original malconformations, or accidental injuries from birth; and, 2. Actual diſeaſes. Amongſt other accidents of the firſt of theſe claſſes, he ſpeaks of the inverſion of the tongue with which children are ſometimes born, or which may happen from ſuction; in ſuch caſes convulſions immediateiy enſue, and ſoon after ſuffocation. The diſeaſe is diſcovered by putting a finger into the child's mouth; and the the fatal event can only be prevented by tickling the throat to provoke vomiting.

In treating of the actual diseases, the author observes, that we judge of childrens complaints from the symptoms of quick or oppressed breathing, from the violence and duration of the fits of crying, from the appearance of the eyes and countenance, much more than from the frequency of the pulse. Cholic shews itself by the suddenness of its attack, by the state of the belly, frequently by exciting vomiting, and by the well-known symptoms in children of pulling up the feet and legs towards the belly.

Mr. Hamilton speaks of the *red gum*, which is distinguished from the measles by the absence of measly symptoms and time of attack, and which easily gives way to a cooling regimen; of the *yellow gum*, which depends on the increased secretion of bile from the change in the circulation through the liver, and requires a similar treatment as in adults; of the *thrush*, or *sprue*, as it is vulgarly called in Scotland, a disease, the nature of which is little understood, and which is often injudiciously treated. We are cautioned against the use of lotions in the early stage of it. Treating of *dentition*, he observes, that in general weakly children cut their teeth teeth later than thofe who are ftronger and more thriving. He recommends the providing the child with fomething which can be fafely applied to his mouth to prefs his gums againft, as often as he is urged to it, as by that means uneafy itching will be gratified, and a gentle flavering, which is always falutary, will be promoted. For this purpofe he prefers a bit of liquorice root, frequently renewed (as it becomes dry and hard) to coral or other hard fubftances, which not only endanger bruifing the inflamed gum, but the thrufting out of thofe teeth that are already formed.

The work clofes with fome remarks on the qualifications of a good midwife, and with a number of ufeful *formulæ* for women and children, and directions for preparing a variety of drinks and food.

